# ImageNet-trained deep neural networks exhibit illusion-like response to the Scintillating grid

**DOI:** 10.1167/jov.21.11.15

**Published:** 2021-10-22

**Authors:** Eric D. Sun, Ron Dekel

**Affiliations:** 1 Mather House, Harvard University, Cambridge, MA, USA; 2 Department of Neurobiology, Weizmann Institute of Science, Rehovot, PA, Israel

**Keywords:** visual illusion, Scintillating grid illusion, deep neural network, computer vision

## Abstract

Deep neural network (DNN) models for computer vision are capable of human-level object recognition. Consequently, similarities between DNN and human vision are of interest. Here, we characterize DNN representations of Scintillating grid visual illusion images in which white disks are perceived to be partially black. Specifically, we use VGG-19 and ResNet-101 DNN models that were trained for image classification and consider the representational dissimilarity ($L^{1}$ distance in the penultimate layer) between pairs of images: one with white Scintillating grid disks and the other with disks of decreasing luminance levels. Results showed a nonmonotonic relation, such that decreasing disk luminance led to an increase and subsequently a decrease in representational dissimilarity. That is, the Scintillating grid image with white disks was closer, in terms of the representation, to images with black disks than images with gray disks. In control nonillusion images, such nonmonotonicity was rare. These results suggest that nonmonotonicity in a deep computational representation is a potential test for illusion-like response geometry in DNN models.

## Introduction

Given sufficient training data, deep neural network (DNN) models are capable of matching human accuracy in challenging image classification tasks (Krizhevsky et al., 2012; He et al., 2015; LeCun et al., 2015; Schmidhuber, 2015; Serre, 2019). Motivated by this recent progress in the field of computer vision, DNN models trained for image classification have started to receive significant attention in neuroscience research (Majaj & Pelli, 2018; Turner et al., 2019). For example, some DNNs have been proposed as models of human shape recognition (Kubilius et al., 2016; Jozwik et al., 2017), visual perceptual learning (Cohen & Weinshall, 2017; Wenliang & Seitz, 2018), and visual crowding (Volokitin et al., 2017) among other aspects of human visual perception (Dekel, 2017; Geirhos et al., 2018; Gruber et al., 2018; Zhang et al., 2018; Kim et al., 2019; Ward, 2019; Lotter et al., 2020), possibly with the exception of more “global” Gestalt effects (Baker et al., 2020). In addition, multiple studies observed that image representation in different stages of the DNN computational hierarchy (i.e., the “features”) have unprecedentedly strong correlation with neural activity at different visual areas in primate cortex (Cadieu et al., 2014; Khaligh-Razavi & Kriegeskorte, 2014; Yamins et al., 2014; Güçlü & van Gerven, 2015; Kriegeskorte, 2015; Yamins & DiCarlo, 2016; Martin Cichy et al., 2017; Grossman et al., 2019). Although the matter is still under debate (Majaj & Pelli, 2018; Doerig et al., 2019, 2020), some aspects of biological vision appear to be well-explained by DNN models trained for image classification despite the two computational architectures being only partially similar (Richards et al., 2019).

Of particular interest in the study of both biological and computer vision is an investigation of the inputs for which the system produces incorrect results. In humans, cases where perception deviates from physical reality, often referred to as visual illusions, have been investigated since antiquity (Gregory, 1968, 1991; Eagleman, 2001). Among other discoveries, research of visual illusions has led to the widespread notion that perception is the consequence of a statistical inference process, and consequently, that many visual illusions reflect statistical assumptions about the visual input (Gregory, 1980; Howe & Purves, 2005; Clark, 2013; Summerfield & Lange, 2014). A natural question is whether visual illusions are shared between biological and computer vision. A few recent studies suggest that there are indeed similarities. Adversarial examples designed to transfer across DNN models were shown to have partial transfer to time-limited human observers (Elsayed et al., 2018; Zhou & Firestone, 2019). Also, biases consistent with classic visual illusions were found in the learned representation of standard DNN models trained for image classification (Ward, 2019), video and motion prediction (Watanabe et al., 2018; Lotter et al., 2020), image denoising (Gomez-Villa et al., 2019), and in orientation representation (Benjamin et al., 2019; Henderson & Serences, 2021); and deep learning has been used to model natural image patch statistics in visual illusions (Hirsch & Tal, 2020). Of course, in humans, bias in a task or a change in neural activity are not necessarily indicative of a change in the perceptual experience (Crick & Koch, 1995; Morgan, 2014; Witt et al., 2015; Linares et al., 2019; Borowski et al., 2019). Human visual experience provides internal representations that can be quantified, and these quantifications may be comparable with those of DNNs. For this reason, we refer to the DNN effect considered in this work as a test of illusion-like response geometry.

**Figure 1.**
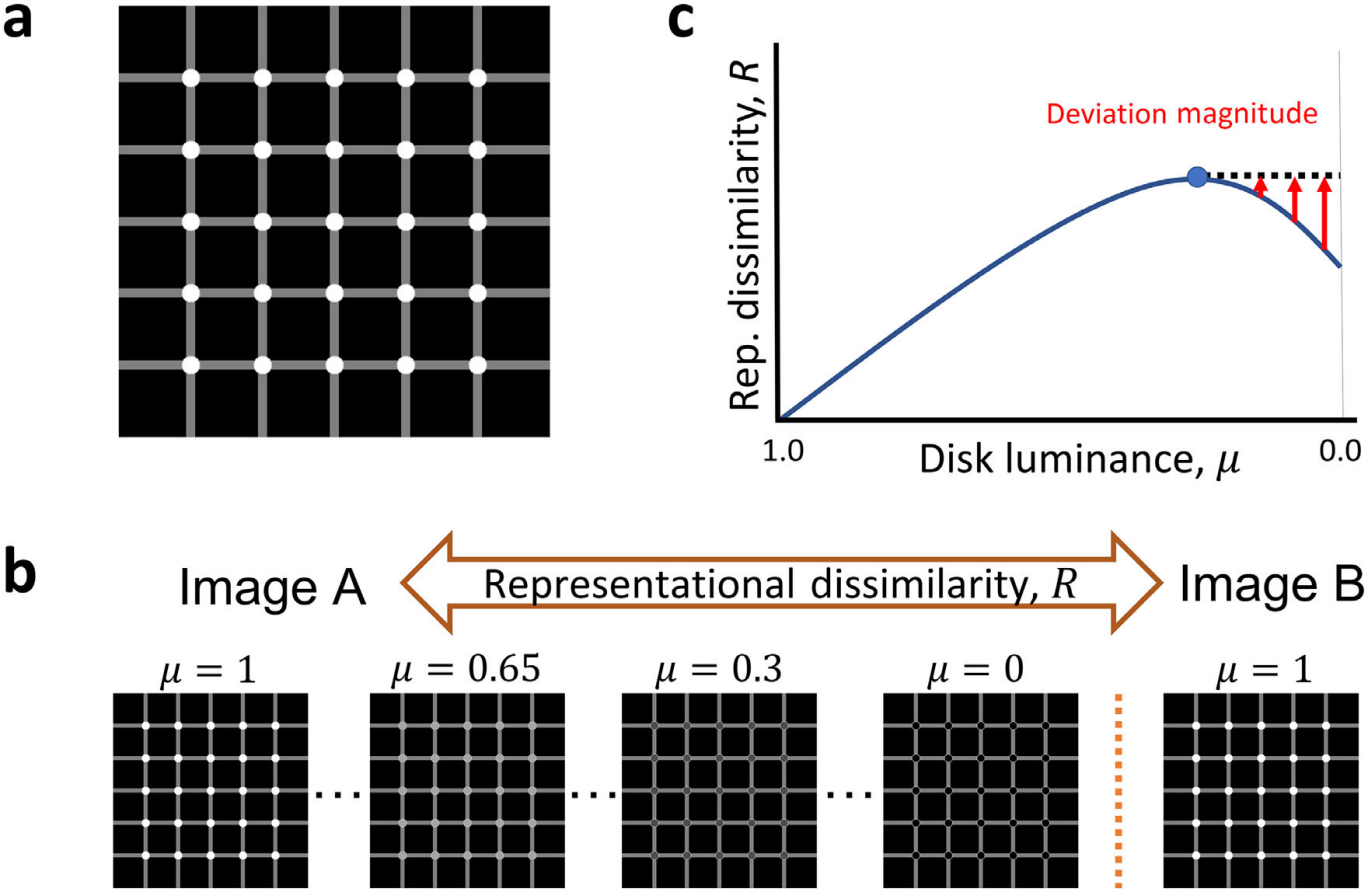
Scintillating grid stimulus and experimental methods. (a) The Scintillating grid visual illusion exhibits illusory scintillation of black smudges within the white grid disks. (b) Schematic representation of the experimental setup. Representational dissimilarity, denoted as R, was calculated as the mean-normalized $L^{1}$ distance of the VGG-19 representation (layer fc8) between two images. One image had a masking region of varying luminance (from μ=1.00 through μ=0.00 by Δμ=-0.05) and the other image was constant with a white-masked region (μ=1.00 throughout). For the Scintillating grid (panel a), the masking region was the grid disks. (c) The magnitude of deviation from a monotonic relation between R(μ) and μ (“deviation magnitude”), d(μ), is calculated as the difference between R(μ) and the maximum R for all luminance values greater than (to the left of) the current value of μ (see Methods).

Here, we focus on a single visual illusion: the Scintillating grid (Figure 1a). In the Scintillating grid, scintillating illusory smudges are perceived within disks located at the intersections of a grid of bars (Bergen et al., 1985; Schrauf et al., 1997). In the classic variant used here (Figure 1a), the disks are white, the bars are gray and the background is black, leading to scintillating illusory smudges that appear black. We investigated the representation of Scintillating grid images for two DNN models trained in image classification: VGG-19 and ResNet-101. Since the Scintillating grid illusion manifests as illusory black smudges inside of white disks, we took Scintillating grid images (as well as control images with reduced or no illusion), and manipulated the luminance inside the disks (or inside a relevant masking region) from white to black (Figure 1b). For an illusion-like DNN representation, the white disk image would be more “similar” to the black disk image than some intermediate gray disk images (Figure 1c) exclusively for the Scintillating grid stimuli. Formally, this means that the representational dissimilarity ($L^{1}$ distance in representation), as a function of luminance, would be nonmonotonic mostly in the Scintillating grid images. The results agreed with this hypothesis, showing significant nonmonotonic trends between representational dissimilarity and luminance in the Scintillating grid images as compared to control images. The nonmonotonic representational dissimilarity also correlated with Scintillating grid perception data obtained from human observers across several setups.

## Methods

### DNN models

We considered two standard ImageNet-trained DNN models in this work: VGG-19 (Simonyan & Zisserman, 2014) and ResNet-101 (He et al., 2016). We used the standard versions of both models, accessed through the Deep Learning Toolbox of MATLAB. The models were trained for classification on 1,000 image classes containing approximately 1.3 million images from ImageNet (Deng et al., 2009). We selected VGG-19 and Resnet-101 because, despite being designed for object recognition tasks, both are top models on Brain-Score (seventh and ninth out of 148 models, respectively, as of July 28, 2021), which ranks computer vision models by their similarity to the brain across several standardized benchmarks (Schrimpf et al., 2018, 2020). In Brain-Score, VGG-19 had higher scores for V1 and V4 while Resnet-101 had the higher score for behavior. The two models are structurally different in that Resnet-101 has a deeper architecture facilitated by residual blocks with identity-like mappings (He et al., 2016). In this work, we mainly focused on the VGG-19 model. Although VGG-19 achieves a lower classification performance compared with newer models such as ResNet-101, it is consistently found to be a promising model correlate of human perception (Kubilius et al., 2016; Dekel, 2017; Gruber et al., 2018). Most analyses were performed on the output representation of the penultimate layer (i.e., the last fully connected layer, such as fc8 in VGG-19), because this computational stage seems to be frequently found as the most similar to different aspects of visual perception (Kubilius et al., 2016; Jozwik et al., 2017), although not always (Dekel, 2017; Grossman et al., 2019).

### Image stimuli

We used several sets of images, each corresponding with a different illusion or control condition (examples shown in Supplementary Figure S1). For each image, a masking region is defined, whose luminance is manipulated as described in the next section. All image sets have been made available on the public Github repository: https://github.com/sunericd/dnn-illusion. Images were created using a canvas size of 768×768 pixels, and then resized to 224×224 pixels to conform to the VGG-19 and ResNet-101 input requirements. Element sizes are reported for the 224×224 pixels image.

#### Scintillating grid (illusion)

Images were defined by variations on three components: bars, disks, and global translation. Bars had a width of 5 pixels and a luminance of 0.5 (on a scale of 0 to 1). The number of bars was the same for horizontal and vertical bars and between 2 and 6 (N×N where N∈[2,3,4,5,6]; overall 5 selections). Disks were positioned at the intersections of the bars and had diameters of 9, 11, or 13 pixels (3 selections). The disks defined the masked region where luminance is manipulated (see *Sinusoid bars (reduced illusion)*). Bar positions had a fixed separation and a varying offset from center of −90, −45, 0, +45, or +90 pixels, separately for vertical and horizontal translations, which amounted to 25 unique translation variations. The background was black (luminance of 0). Overall, 375 variants were used. In the main experiments, we used a 1-pixel disk border with luminance 0.8 for the purpose of preserving disk shape when the disk luminance changes. Using an edge of 1 pixel is reasonable under the assumption that the black smudges are slightly smaller than the disks (e.g., have a radius of 90% of the disks; current behavioral estimates suggest 80% with exact values depending on parameters; Matsuno & Sato, 2019). As evident by inspecting Figure 1a, introducing the 1-pixel disk border preserves illusion perception (the standard Scintillating grid has unbordered disks). In fact, all 375 variants had intact illusion perception under standard viewing conditions. In addition to the 1-pixel disk border experiments, we considered additional experiments with disk borders of 0 pixels (“unbordered”), 2 pixels, or 3 pixels (Results in Supplementary Figure S10).

#### Sinusoid bars (reduced illusion)

When grid bars are not straight, the illusory effect is reduced for both the Hermann grid (Schiller & Carvey, 2005; Geier et al., 2008) and the Scintillating grid (Levine & McAnany, 2008). Here, we used bars with a sinusoidal curvature, having a sine amplitude of 3.5 pixels, and a wavelength matched to the bar separation (see Supplementary Figure S1). Each sinusoid bars reduced illusion image was matched with an image from the Scintillating grid image set, with the exception of the grid bars being sinusoidal instead of straight.

#### Large disks (reduced illusion)

The stimuli were identical to the Scintillating grid condition, but with double disk size variants (i.e., 18, 22, or 26 pixels). It is evident that, under standard viewing conditions, illusion perception is significantly reduced, if not entirely absent (see Supplementary Figure S1) (Schrauf et al., 1997; Sun, 2019). Each image in the large disks set had the same parameters as a corresponding Scintillating grid image, with the exception of larger grid disks.

#### Offset bars (control)

Illusion perception is decreased and possibly eliminated when the grid bars are translated such that their intersections do not align at the disks (Supplementary Figure S1) (Schrauf et al., 1997). We used such images as an additional control. Each image in the offset bars decreased illusion set had the same parameters as a corresponding Scintillating grid image, with the exception of the grid bars, which were translated such that every disk was equidistant from its four neighboring bars.

#### No bars (control)

Removal of the grid bars decreases and possibly eliminates perception of the scintillating illusion (Supplementary Figure S1) (Schrauf et al., 1997). As such, we used a no bars control image set that was matched with the illusion image set. Each no bars image had the same image parameters as its corresponding Scintillating grid image with the exception of the grid bars, which were removed.

#### Disk-masked natural (control)

To investigate the consequence of our disk masking procedure in a more natural setting, we used 375 images randomly selected from the first 1,000 validation images of ImageNet ISLVRC. We applied the same disk masking as in the Scintillating grid. The number and position of disks were selected using the same set of variants as used for the Scintillating grid.

#### Pixel-masked natural (control)

Because the white regions in natural images are typically not organized as a perfect grid, we considered a control whereby the 10% highest luminance pixels in the natural image were used as the masking pattern instead of the disk mask in other image sets. We applied this image-specific masking to each of the 375 aforementioned natural images.

#### Number of disks (control)

Stimuli were identical to the Scintillating grid set with a 5×5 grid (providing 75 variants, unlike the other image sets each having 375 variants), but having the number of the white disks decreasing from 25 to 0 instead of the disk luminance decreasing from 1 to 0.

#### Gray background (control)

To control for contrast effects, we designed images with Scintillating grid disks on a gray (luminance 0.5) background with no bars. Each gray background image had the same image parameters as its corresponding Scintillating grid image. Results and discussion for the gray background control is included in Supplementary Figures S9, S10, S11, and Appendices B and C.

### DNN experimental setup

We used two experimental setups. In the luminance setup, a masking region was defined for each image of each set. With the exception of the pixel-masked natural image set (described elsewhere in this article), the masking region corresponded with the inner region of the disks. The luminance of the masking region was changed between white (μ=1.00) and black (μ=0.00) along 21 uniformly spaced luminance levels (Δμ=-0.05). These differently masked images were compared using representational dissimilarity (described elsewhere in this article) defined with respect to the “reference” image with a white mask (μ=1.00).

In the “number of disks” setup, the number of white disks was changed instead of disk luminance. Images with a different number of white disks were compared using representational dissimilarity to the reference image, which had all white disks. Note that the reference images in the “number of disks” setup are identical to the reference images in the luminance setup for the Scintillating grid image set (i.e., the original illusion; Figure 1a).

Representational dissimilarity quantifies how dissimilar two images are in terms of the DNN representation. Specifically, to compare an image A(q) with the reference image $A^{\mathrm{ref}}$, we first record the values in a given computational stage for the two images, resulting in two tensors a(q) and $a^{\mathrm{ref}}$, each having dimensions of M×N×K values (rows, columns, and convolution kernels, respectively). The computational stage considered in most analyses is the post-ReLU penultimate layer (i.e., fc8 in VGG19). Next, we compute the $L^{1}$ distance between these tensors:

(1) $$r_{L1}\left(q\right)=\sum_{m=1}^{M}\sum_{n=1}^{N}\sum_{k=1}^{K}\left|a{\left(q\right)}_{mnk}-a_{mnk}^{\mathrm{ref}}\right|$$

Finally, we normalize $r_{L1}$ by its mean across different values of q (where q is the luminance level or the number of disks):

(2) $$R\left(q\right)=\frac{r_{L1}\left(q\right)}{\bar{r_{L1}}}$$

We verified that using the $L^{2}$ metric instead of the $L^{1}$ metric for representational distances led to very similar results (Supplementary Figure S3). We decided to not use the cosine distance as a metric for representational dissimilarity, because it would be insensitive to differences in absolute magnitudes between the two representations, which are of interest here.

### Proposed test of illusion-like response geometry: Deviation magnitude

We suggest the magnitude of deviation from monotonicity in the DNN representation as a model correlate of illusion perception. Specifically, we compute the deviation from monotonicity at each disk luminance as the non-negative difference between a given R and the maximal value of R in all higher luminance levels (Figure 1c). We refer to this measurement as the deviation magnitude. The motivation for this metric is that, in the absence of illusion perception, loss of contrast, or changes in shape contours, the representational dissimilarity, R, may be speculated to increase monotonically for decreasing luminance levels since the pixel difference between the two images would increase. In the presence of illusion, the black disks are “similar” to the white disks, which implies that R should be nonmonotonic (R between white and some intermediate luminance to be larger than R between white and black).

### Human experimental setup

Both authors scored the magnitude of the scintillating illusion in random images over all luminance levels for one variant of the Scintillating grid, one variant of the no bars control, and one disk-masked natural image. Each image variant was scored five times by each observer. The observers used a scale of 0 to 5 to indicate increasing illusion magnitude, which is similar to previous scoring methods for Scintillating grid illusion strength (Schrauf et al., 1997). Observers were seated approximately 50 cm from the display, in an otherwise uncalibrated setting. Image dimensions were displayed at 10 × 10 cm (approximately 11.4 visual degrees) on a gray background (luminance of 0.5). The stimuli were presented on a display monitor from a Dell XPS 13 9360 computer. The illuminance of the screen during the experiment ranged from 140 to 160 lux when the stimuli was presented and from 20 to 50 lux without stimuli. The illuminance of the room ranged from 10 to 80 lux.

## Results

### Disk luminance experiment

When the Scintillating grid disk luminance is decreased from white to black, the pixel distance, relative to a reference image with white disks, increases. However, in human vision, the white disks in the intersections of the Scintillating grid are perceived as black regions superimposed on the disks and are therefore more visually similar to black disks than to gray disks. Here, we hypothesized that the DNN models would exhibit illusion-like response geometry, characterized by white being more “similar” to black than to gray in the DNN representation for disks positioned in the bar-intersections of Scintillating grid images. To test this, we considered one illusion image set, three sets of images with reduced illusions, and three nonillusion control image sets (see Methods). According to our hypothesis, the illusion images would produce the greatest nonmonotonic relationship between representational dissimilarity (R) and disk luminance (μ) (Figure 1c). In the control image sets, we hypothesized that the representational dissimilarity R would monotonically increase from R=0 at μ=1 to $R_{\max}$ at μ=0, mainly owing to linear increases in the pixel distance between the input images.

**Figure 2.**
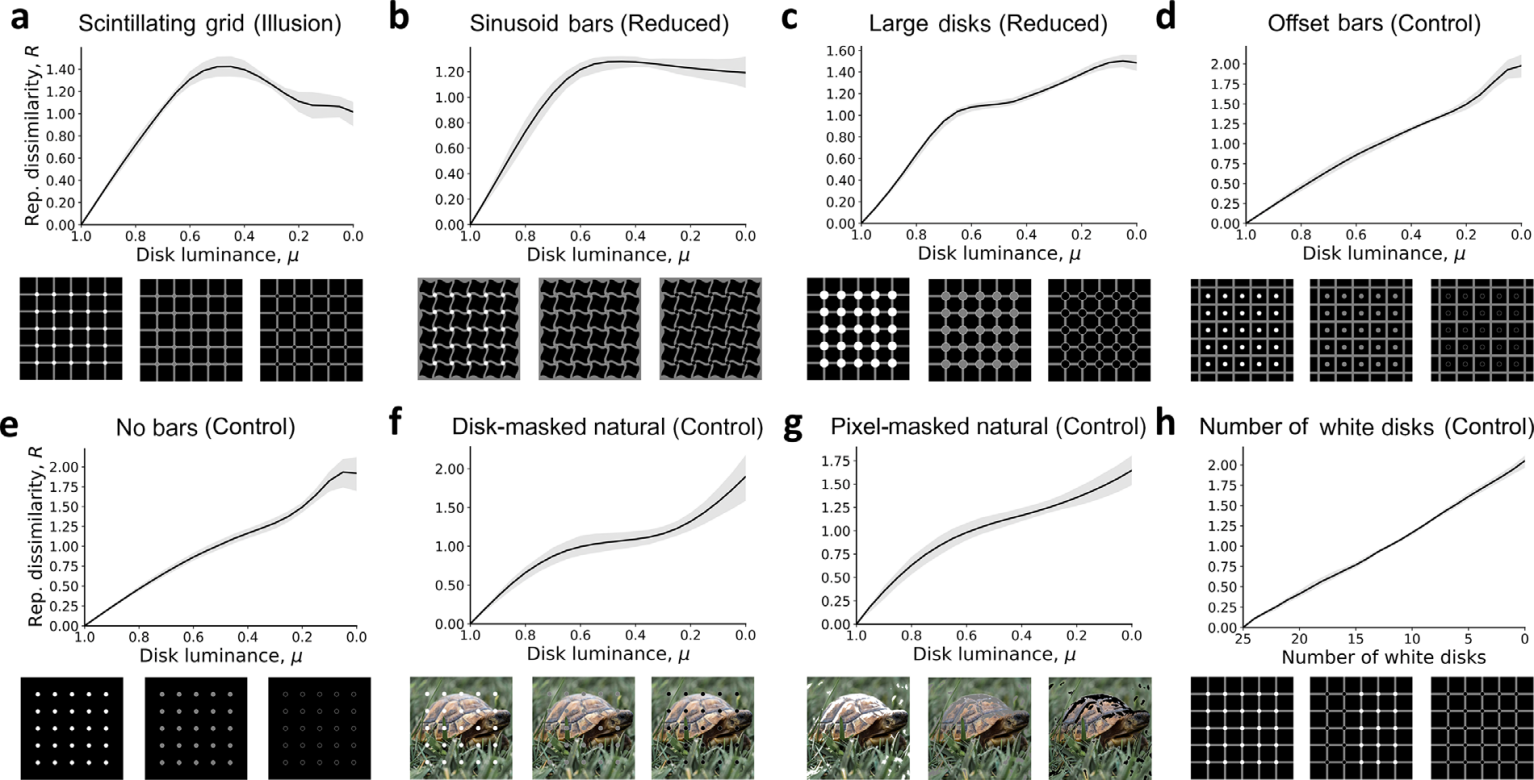
Nonmonotonicity of representational dissimilarity in Scintillating grid and controls. (a–g) Shown is the mean representational dissimilarity, R, for decreasing disk luminance, μ, calculated as described in Figure 1. Shaded regions correspond to the interquartile range. (a) For Scintillating grid illusion images. (b–c) For variants of the Scintillating grid with reduced illusory perception in human: (b) sinusoid bars and (c) large disks. (d–g) For controls having virtually no illusion perception in human: (d) offset bars, (e) no bars, (f) disk-masked natural images, and (g) pixel-masked natural images (see Methods). Here, the example natural image is represented by a public-domain image instead of ImageNet images. (h) For a control setup in which the number of the white disks is decreased instead of the disk luminance. Grids with progressively fewer white disks were compared with the full white-disk grid. Overall, results showed that the non-monotonicity in the relation between R and μ was strong for Scintillating grid images (a), weak for reduced illusion images (panels b–d), and absent, on average, for nonillusion controls (e–h). Note that the *y*-axis scaling is different in the different panels.

**Figure 3.**
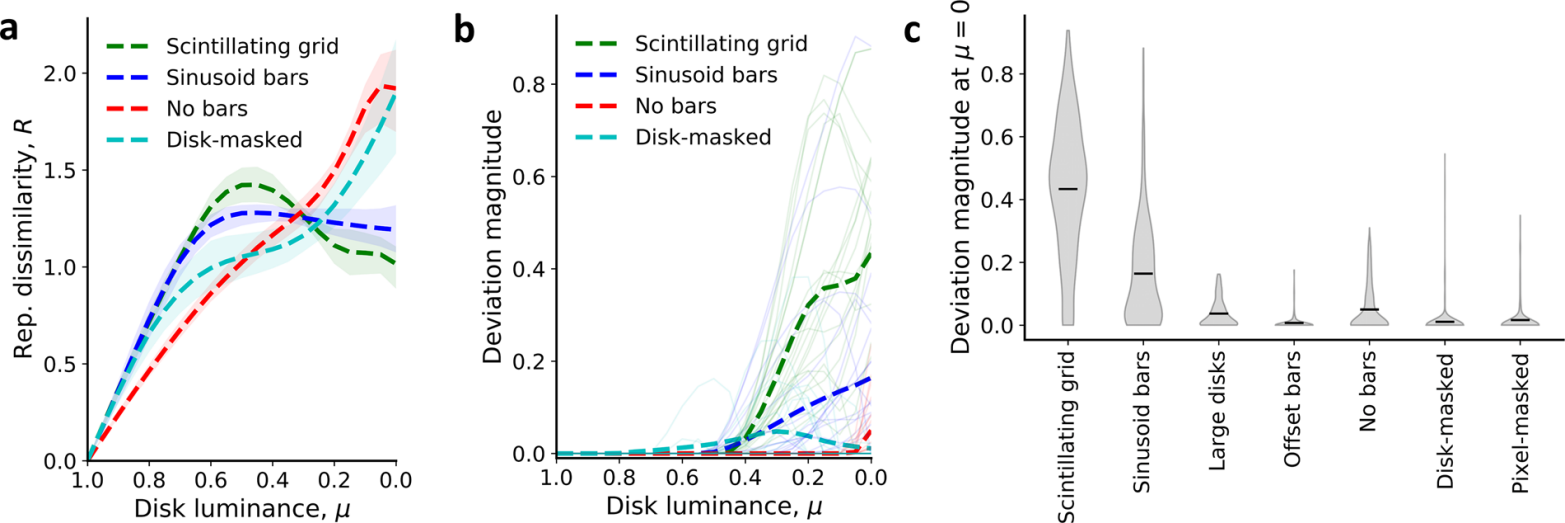
Deviation from monotonicity in the relation between representational dissimilarity R and disk luminance μ. (a) Representational dissimilarity averaged across all 375 image variants in four stimuli sets: Scintillating grid, sinusoid bars, no bars, and disk-masked natural images (reproduced from Figure 2a,b,e,f). Shaded region represents the interquartile range. (b) The mean deviation magnitude d (see Figure 1c), as a function of disk luminance, for Scintillating grid, sinusoid bars, no bars, and disk-masked natural images. Deviation magnitude is measured as the difference between R(μ) and the maximum R for all disk luminance greater than the current μ; it is a measure of the deviation from a monotonic relation between R(μ) and μ. Solid lines show 20 randomly selected individual measurements. (c) Deviation magnitudes, d, at disk luminance μ=0 for all image variants. Results showed that deviations for Scintillating grid images were significantly greater than those of other images. On average, reduced illusion images (sinusoid bars, large disks) produced larger deviations than nonillusion images (offset bars, no bars, and natural images).

Consistent with our hypothesis, in the Scintillating grid images, there were significant deviations from the monotonic relation with decreasing disk luminance (Figure 2a). The representational dissimilarity increased in a monotonic fashion to a maximum at μ=0.45 before decreasing and leveling off for lower luminance levels (μ<0.45). Variants of the Scintillating grid with reduced illusion perception in humans (e.g., sinusoid bars, large disks) (Schiller & Carvey, 2005) exhibited a lesser degree of non-monotonicity (Figure 2b–d). This trend was noticeably different from the completely monotonic behavior observed in control images (Figure 2e–g). On average, the Scintillating grid images produced a more nonmonotonic relation between R and μ than either reduced illusion or nonillusion control images (Figure 3a). These trends were observed for different grid sizes, different numbers of disks, and different combinations of vertical and horizontal translations (see Methods). Results using the non-normalized $L^{1}$ representational dissimilarity are shown in Supplementary Figure S2.

To quantify the magnitude of this illusion-like, nonmonotonic effect in VGG-19 representational dissimilarity, we computed the deviation magnitude d at each experimental interval (see Methods for details). In the Scintillating grid images, low deviation magnitudes were observed for high luminance (μ>0.45), but significantly increased for lower luminance (μ<0.45) (Figure 3b). In comparison, the reduced illusion and non-illusion control images produced minimal deviation magnitude throughout most disk luminance levels (Figure 2b). Compared with every other class of images, the Scintillating grid illusion images observed significantly higher average deviation magnitude at μ=0 (Figure 3c, $p<10^{-54}$ for all Mann–Whitney *U* tests between mean Scintillating grid d(μ=0) and the mean d(μ=0) of reduced illusion images and of nonillusion control images). Similar results were observed for the Resnet-101 DNN ($p<10^{-110}$; Supplementary Figure S5), and for VGG-19 when using $L^{2}$ representational distances instead of the $L^{1}$ metric ($p<10^{-55}$; Supplementary Figure S3). Compared with VGG-19, ResNet-101 produced a slightly larger difference between Scintillating grid and sinusoid bar deviation magnitudes and a lower deviation magnitude for disk-masked natural images (Supplementary Figure S5). We also explored disk parameterizations from white to black through color space, which although less intuitive, produced similar results (Supplementary Figure S6). Moreover, in Appendix B, we consider manipulations of background luminance and disk border width, which suggest that the nonmonotonicity observed here is better explained by illusion perception than by loss of contrast or shape, which occurs when the disks are the same luminance as the bars.

### Number of black disks experiment

Instead of manipulating the disk luminance, we considered manipulating the number of white disks. In this setup, the white disks in the Scintillating grid were progressively replaced with black disks, and compared with the original Scintillating grid image (the reference) (Figure 2h). Note that, similar to increasing disk luminance in the previous section, increasing the number of white disks should linearly increase the pixel distance between the two compared images. However, unlike the previous setup, the number of white disks is also proportional to the magnitude of the perceived illusion for human observers given that each white disk contributes equally to the illusion effect, which is localized at the disks. Therefore, if the observed nonmonotonicity indicates an illusion-like response, then varying the number of white disks in the Scintillating grid should result in a linear relation with representational dissimilarity (R), which was indeed observed (Figure 2h). Given that this control relies on a linearity assumption, we separately manipulated each disk from white to middle grey (μ=0.5) and finally to black and recovered a nonmonotonic relation between the number of changes and the representational dissimilarity (see Supplementary Figure S8).

### Origin and propagation of illusion-like deviation

**Figure 4.**
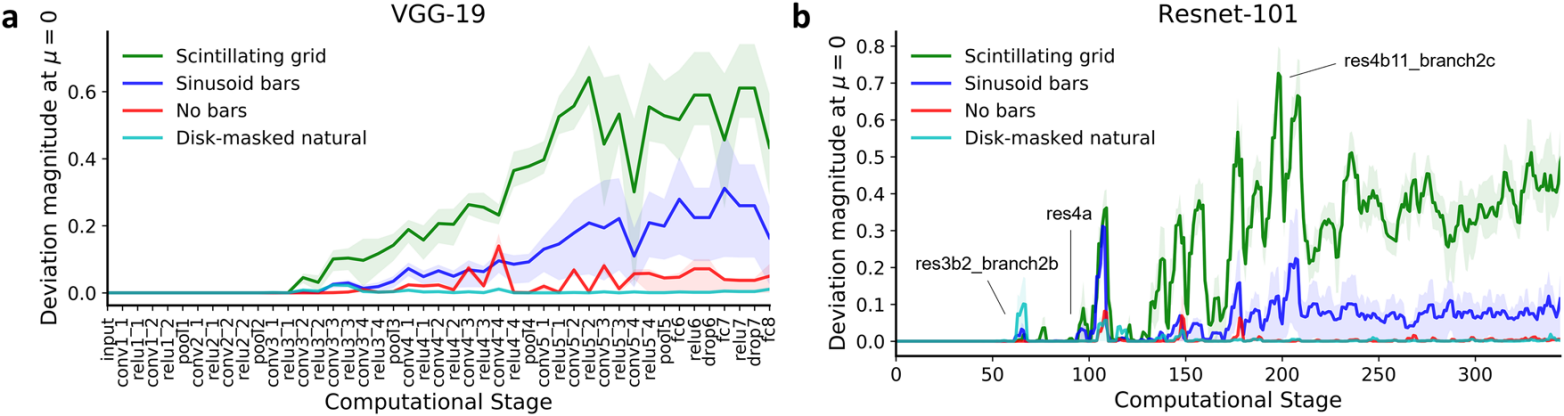
Deviation magnitudes across the network hierarchy. Shown, for each computational stage of the (a) VGG-19 model and (b) ResNet-101 model, is the mean deviation magnitude at μ=0. Deviation magnitudes were averaged over all relevant images and the shaded region represents the interquartile range. By “computational stage,” we refer to intermediate computational stages that compose the network layers (e.g., VGG-19 has 19 layers, composed of 45 computational stages that we show). Results for both models show gradual increase in deviation magnitudes in intermediate computational stages.

Thus far, we have been concerned with deviation magnitude measurements in the penultimate layer (e.g., fc8 in VGG-19). We next considered all intermediate computational stages, a term we use to refer to layer outputs as well as intermediate layer computations (e.g., before and after ReLUs). This analysis provided a foundation to speculate which computational stages are responsible for the origin of the non-monotonicity. Results, in both VGG-19 and ResNet-101 (Figure 4), show close to zero deviation magnitude in the early layers, a gradual increase in deviation magnitudes in the intermediate layers, and a plateau in subsequent layers. These measurements are consistent with an origin of the nonmonotonicity in multiple intermediate layers followed by propagation of nonmonotonicity to the penultimate layer. Specifically, in VGG-19 (Figure 4a), the Scintillating grid results showed induction of nonzero deviation magnitude at conv3_2, an increase in deviation magnitudes over subsequent stages until relu5_2, and no further increase until the penultimate fc8 layer. In ResNet-101, the Scintillating grid showed nonzero deviation magnitudes as early as res3b2_branch2b, with generally increasing deviation magnitude starting at res4a and stopping around res4b11_branch2c, the computational stage with maximum deviation magnitude. After res4b11_branch2c, the deviation magnitude approximately plateaus until the penultimate layer (Figure 4b). The maximum deviation magnitude occurred at res4b11_branch2c. The sinusoid bars image set, for both VGG-19 and ResNet-101, showed deviation magnitudes similar to the Scintillating grid but weaker in magnitude, consistent with reduced but nonabsent illusion perception (Figure 4a,b). The no bars and disk-masked natural nonillusion controls showed almost zero deviation magnitudes in all computational stages, consistent with absence of illusion perception. Overall, the results suggest that intermediate computational stages are primarily responsible for the illusion-like response in VGG-19 and ResNet-101. This observation is perhaps in line with an intermediate (postretinal) origin of the Scintillating grid illusion in humans (see Discussion). There were also significant differences between the deviation magnitudes of the Scintillating grid and gray background control images across both DNN hierarchies, which support an illusion-specific DNN representation for the Scintillating grid that is not fully explained by contrast or shape loss (see Supplementary Figure S11 and Appendix B).

### Comparison with human vision

**Figure 5.**
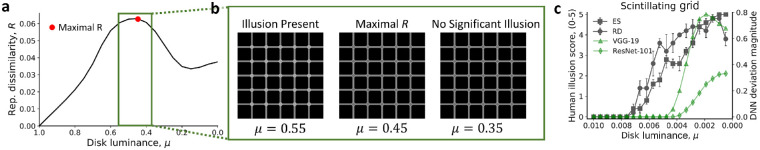
Comparison with human perception. (a) VGG-19 representational dissimilarity R as a function of disk luminance μ for one stimulus (see panel b) in the Scintillating grid image set. The red dot denotes the point of maximum R, which roughly corresponds with the transition from illusion-absent to illusion-present grid images around μ≈0.45. This is apparent by inspection of the Scintillating grid images under standard viewing conditions. (b) Displayed is a Scintillating grid image variant used for analysis in a and c for a range of disk luminance values. (c) Mean illusion scores (0–5) of two human observers (ES and RD), and VGG-19 and ResNet-101 deviation magnitudes for a single variant (depicted in b) from the Scintillating grid image set. Error bars in human illusion scores represent the standard error of the mean (5 samples per disk luminance level).

Because correlations between human and DNN models of vision are of great interest, we sought to directly compare human perception of the Scintillating grid with the DNN representational dissimilarity and deviation magnitude measurements. The standard Scintillating grid stimulus induced a significant illusion-like response in VGG-19 for low to intermediate disk luminance, μ<0.45 (Figure 2a), corroborating loss of illusion perception in humans at intermediate disk luminance (Schrauf et al., 1997; Sun, 2019). This similarity in perceptual ranges can be readily verified by visual inspection of Figure 5a. To strengthen this observation, two observers scored illusion magnitude for the image set shown in Figure 5a. Human-scored illusion magnitudes were similar to the VGG-19 deviation magnitudes for the Scintillating grid in that both values generally increased past an intermediate disk luminance threshold (Figure 5b). A similar consistency was observed in nonillusion control images and reduced illusion control images (see Supplementary). Compared with DNNs, human perception of the Scintillating grid illusion seems to span a slightly broader range of disk luminance values. Mean disk luminance values at half-maximum illusion score or deviation magnitude were μ=0.45 for human observers, μ=0.3 for VGG-19, and μ=0.2 for ResNet-101. Together, these results suggest correlations between VGG-19 representation and human perception of the Scintillating grid illusion. In a second experiment, we directly measured response times in discrimination tasks (as a proxy for representational dissimilarity) between the Scintillating grid and nonwhite disk luminance versions. The results of this second experiment are discussed in Appendix C and provide further support of nonmonotonic or close to nonmonotonic responses to the Scintillating grid in human observers.

## Discussion

Here we report nonmonotonicity in representational dissimilarity as an indicator for illusion-like response geometries of the Scintillating grid in DNNs trained for image classification. Specifically, we found that the DNN image representation is nonmonotonic with respect to disk luminance of Scintillating grid images (Figures 1, 2a, 3). Such nonmonotonicity was much weaker, usually absent, in several controls (Figures 2b–h, 3). For example, the nonmonotonicity was mostly abolished when using large disks or sinusoidal, offset, or absent bars (Figures 2b–e, 3), which have also been shown to reduce illusion perception in human (Schrauf et al., 1997; Levine & McAnany, 2008). Also, the nonmonotonicity was typically absent when manipulating luminance in natural images (Figures 2f–g, 3), which typically do not provide strong illusion perception for human. There were some image stimuli that did not produce an illusion under human observation but had significant nonmonotonicity in DNN representational dissimilarity (see Figure 3b, Supplementary Figure S10). These outlier images may be of interest in dissecting the differences between human and DNN perception of the Scintillating grid. Finally, manipulating the number of white versus black disks instead of luminance led to a linear, monotonic relation between representational dissimilarity and the number of white disks (Figure 2h), suggesting that the nonmonotonicity from manipulating luminance was not the result of pixel differences. We also found an alternative explanation, in terms of loss of contrast or shape, to be less consistent with results from manipulations of background luminance and disk border width (see Appendix B). Overall, these results suggest that nonmonotonicity in DNN representational dissimilarity may indicate an illusion-like response geometry to the Scintillating grid.

A crucial question raised by this work is *why* nonmonotonicity in DNN processing exhibits a response pattern that, to some extent, is similar to human illusion perception. This question is particularly relevant since illusory perception in the Scintillating grid necessitates eye movements (Schrauf et al., 1997) that are obviously absent from the DNN. Perhaps an answer is given by considering the link between competition in a visual representation (Blake & Logothetis, 2002; Gruber et al., 2018) and nonmonotonicity of representational distances. If representational distances are similar in DNNs and in biological vision (Kriegeskorte, 2015), then the DNN nonmonotonicity indicates that the “white disks” version of the Scintillating grid neural representation is similar to the “black disks” version. Hence, Scintillating grid stimuli can be represented by two alternative brain representations, with somewhat higher affinity to the correct “white” version. Such competition, especially in the visual periphery, may lead to alternating periods of perceptual dominance for each alternative (Blake & Logothetis, 2002), resulting in the Scintillating grid illusion. According to this view, the Scintillating grid illusion is explained by the geometry of a learned visual representation. This explanation is thus orthogonal (i.e., at a different level of explanation) to neuronal-level mechanistic accounts (Yu & Choe, 2006). Furthermore, the Scintillating grid has important time-series characteristics that cannot be captured by the DNN models, which use still images as input. Perhaps, the DNN model captures an average characteristic of the time-varying effect: instead of scintillation between illusion-present and illusion-absent time frames in human, the DNN might exhibit the middle-ground of a weak (but fixed) effect. An interesting future direction is to extend the experiments to video-based DNNs (Watanabe et al., 2018; Lotter et al., 2020), which, we suspect, might not be mature enough at present. It would also be interesting to extend this work by applying an adversarial setup (Szegedy et al., 2014; Kurakin et al., 2017; Elsayed et al., 2018; Zhou & Firestone, 2019) to discover images in which manipulations of luminance show nonmonotonic representational distances. Would these images also exhibit a scintillating illusion?

Our findings may be relevant to existing research since the computational and anatomical substrates of the Scintillating grid and other grid illusions are not fully understood (Spillmann & Levine, 1971; Wolfe, 1984; Spillmann, 1994; Schrauf et al., 1997). The Scintillating grid can be regarded as a stronger variant of the classic Hermann grid illusion, in which a somewhat similar effect is evident at the intersections of the bars in absence of any disks (Hermann, 1870; Spillmann, 1994; Schrauf et al., 1997). The classical explanation of the Hermann grid by Baumgartner posits that the illusion is mediated by neurons in the retina with center-surround receptive fields (Baumgartner, 1960, 1990). Baumgartner's theory can, at least partially, apply to the Scintillating grid (Thomson & Macpherson, 2018). However, more recent work using variants of the Hermann grid and the Scintillating grid stimuli suggest that Baumgartner theory alone is not sufficient to explain all grid illusions (Spillmann & Levine, 1971; Wolfe, 1984; Spillmann, 1994; Schrauf et al., 1997). For example, when the bars are distorted, the illusory perception is largely diminished for the Hermann grid and the Scintillating grid (Schiller & Carvey, 2005; Geier et al., 2008; Levine & McAnany, 2008). Such results suggest the involvement of visual processing stages that are downstream to the retina, such as V1. To our knowledge, there is no direct electrophysiological evidence of this claim, which, indeed, may be nontrivial to obtain. Unlike the brain, the DNN models considered here permit easy access to the entire computation hierarchy. Analyzing the deviation magnitude along the computational hierarchy showed the largest deviations from monotonicity in the deep stages of computation, in both VGG-19 and ResNet-101 (Figure 4). Although the computation in human and DNN is not immediately comparable, studies have suggested similarities between early DNN computation and human opponent-color and frequency-selective representations (in retina and in V1), while deeper DNN stages seem to be similar to deeper brain areas, such as V4 or IT (Yamins et al., 2014; Güçlü & van Gerven, 2015; Kriegeskorte, 2015; Cichy et al., 2016; Eickenberg et al., 2017). Consequently, the results using the DNN metrics offered here are more consistent with a cortical (post-V1) origin of the Scintillating grid than an origin earlier in human visual processing, with the later stages of the DNN corresponding most closely to high-level cortical areas. We observed relatively higher layer deviation magnitudes for the sinusoid bars and no bars images in VGG-19 than ResNet-101 (Figure 4), which both have some level of illusion under human observation (Supplementary Figure S7). This difference between the two models may be reflective of VGG-19 generally being regarded as a better approximation for brain cortical processing than ResNet-101 (Schrimpf et al., 2018).

As for extensions of this work to other illusions, note that our approach exploits the non-continuity in the perceptual phenomena (i.e., white disks becoming black, not gray), which is presumably absent in the Hermann grid and some of its analogues. The general framework of identifying an image parameterization corresponding to the illusion effect and then testing for non-monotonicity in DNN representational dissimilarity may be extendable to some other classes of illusions, but in each of these cases, modifications to our current approach will likely be needed.

## Supplementary Material

Supplement 1
