# Supplementary material for: ImageNet-trained deep neural networks exhibit illusion-like response to the Scintillating grid
Source: J Vis. 2021 Oct 22;21(11):15. doi: 10.1167/jov.21.11.15 (PMC8543405; doi:10.1167/jov.21.11.15)
Supplement: Supplement 1 [file jovi-21-11-15_s001.pdf]

Supplementary Material

Appendix A: Supplementary figures

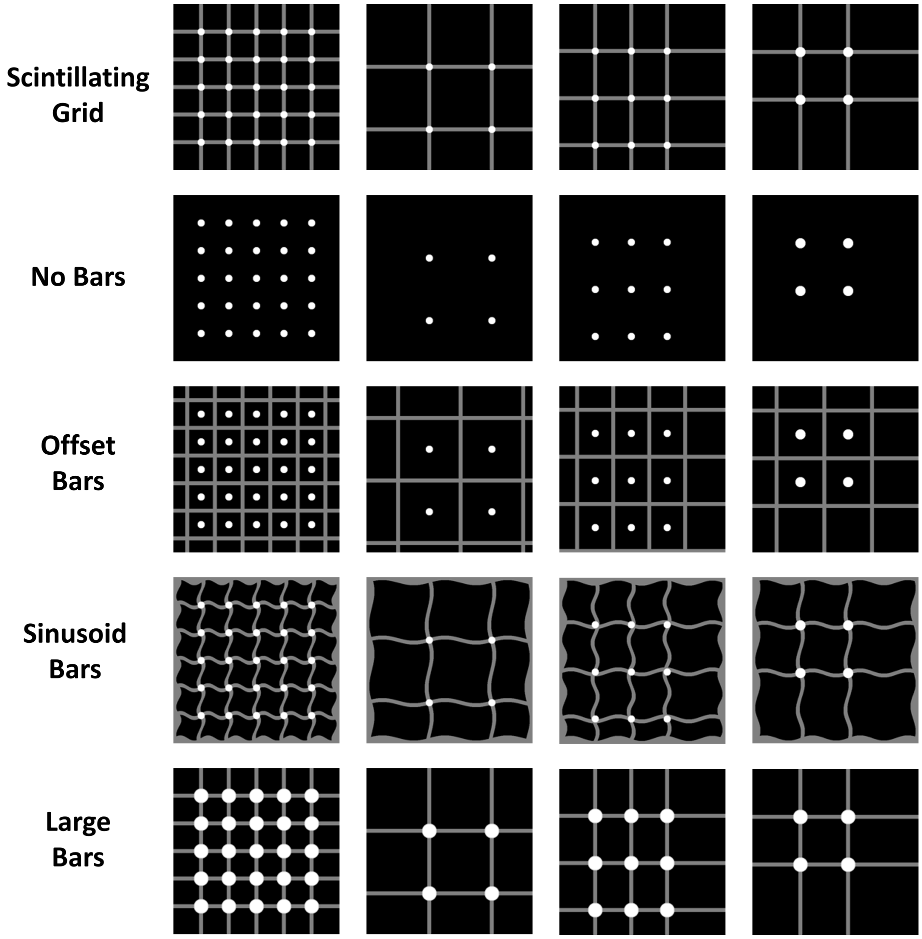

Figure S1: Examples of images used in the experiments. Natural image examples (disk-masked and pixel-masked) are omitted due to copyright concerns.

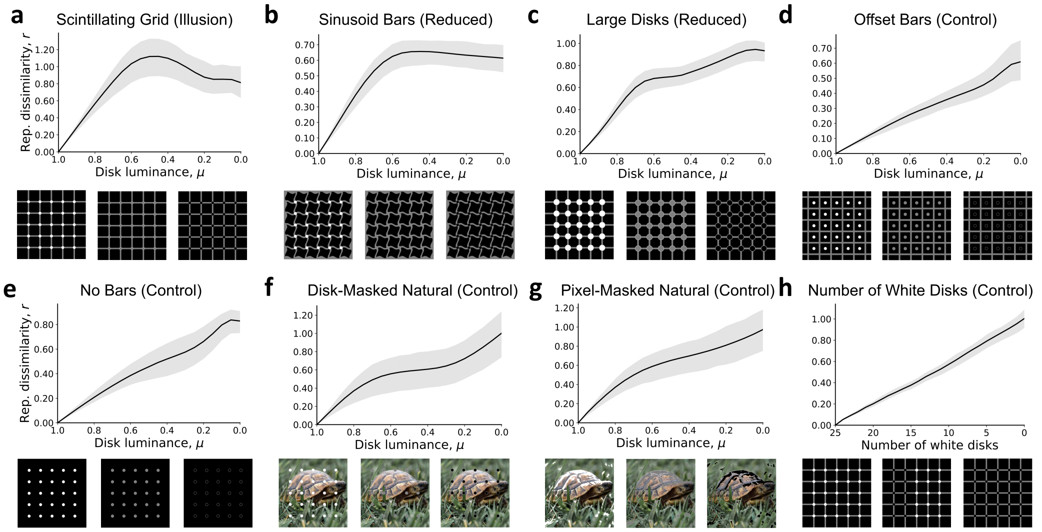

Figure S2: Non-monotonicity of non-normalized representational dissimilarity in Scintillating Grid and controls. (a-h) Non-normalized representational dissimilarity as a function of disk luminance for the same experiments described in Fig. 2.

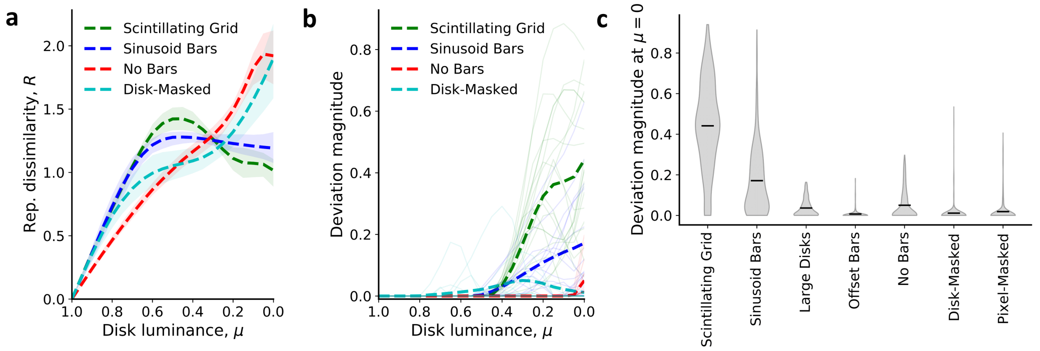

Figure S3: Deviation from monotonicity when using L^2 representational dissimilarities for VGG-19. In all other analyses in this work, we measured representational dissimilarity as the L^1 distance between two VGG-19 representations. The L^2 (Euclidean norm) is another method for quantifying representational distances (see Methods). Panels (a-c) follow Fig. 3, showing the results when representational distances are measured using the L^2 metric instead of the L^1 metric. Results using L^2 distance produced similar results as L^1 (compare with Fig. 3). Namely, the Scintillating Grid images had the greatest non-monotonicity as compared to reduced illusion and non-illusion control images.

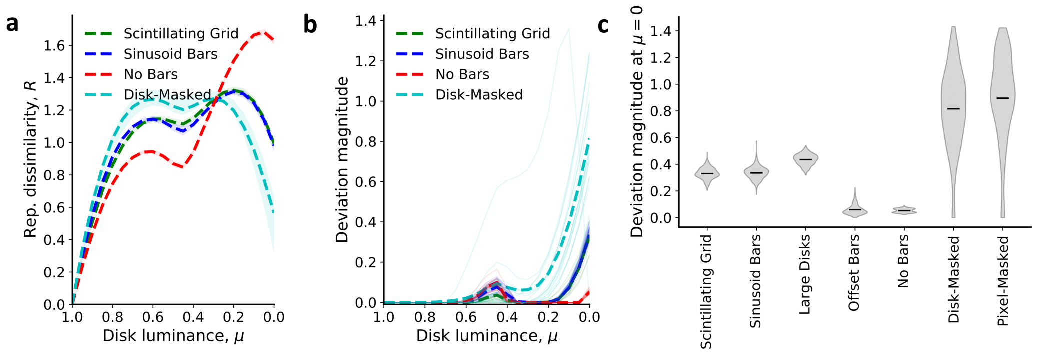

Figure S4: Deviation from monotonicity for weight-scrambled VGG-19 (control). Panels (a-c) follow Fig. 3, showing the results when model connection weights were randomly permuted within layer. Unlike the results with the ImageNet-trained VGG-19 (Fig. 3), these results show deviations from monotonicity for all stimuli sets, i.e. not specific to Scintillating Grid images causing illusion perception in humans.

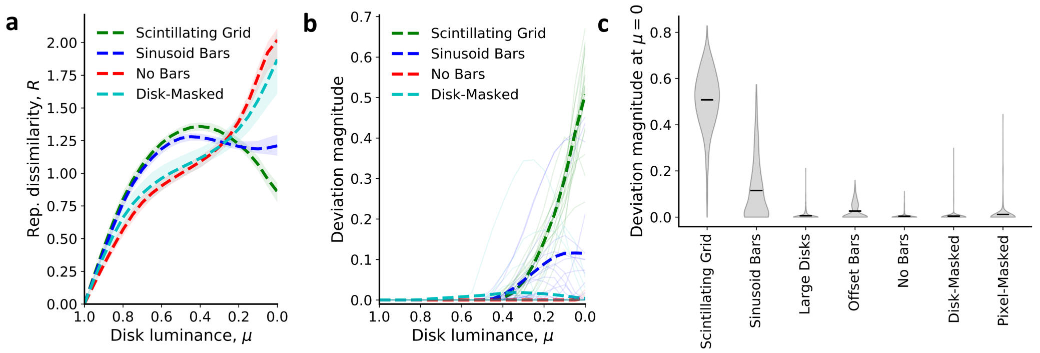

Figure S5: Deviation from monotonicity for ResNet-101. Panels (a-c) follow Fig. 3, showing the results for the ResNet-101 model trained in the same image classification task as the VGG-19 model. Results with ResNet-101 were very similar to the results observed for VGG-19 in the main text (see Fig. 3.

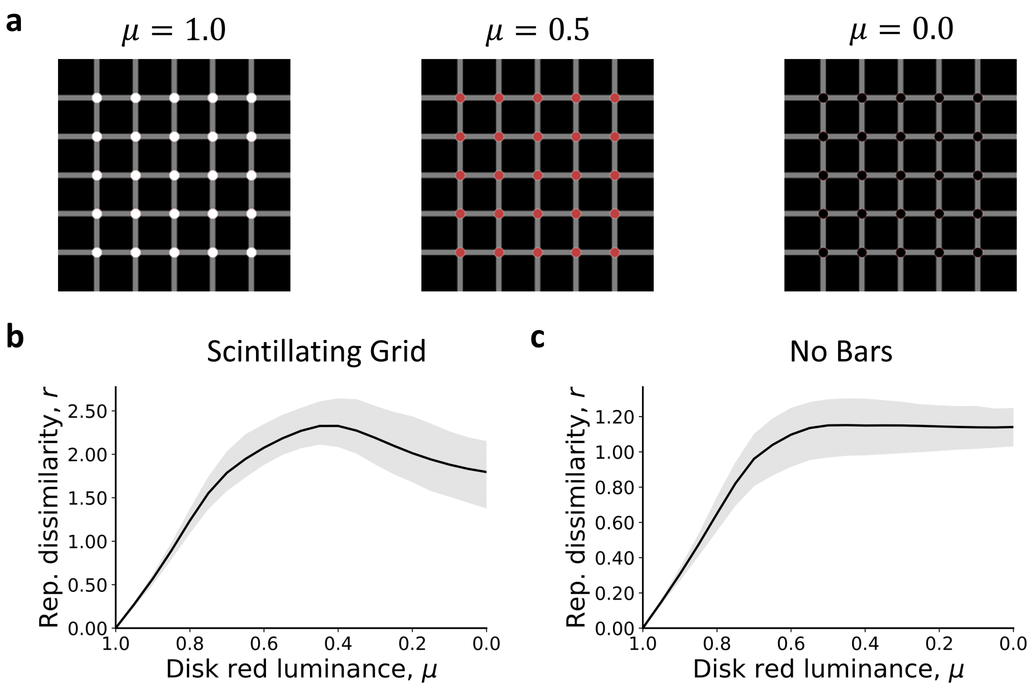

Figure S6: VGG-19 representational dissimilarity for disk parameterization from white to black through red color. Examples of red-disk-parameterized Scintillating Grid image stimuli are shown in (a), and plots of the representational dissimilarity for the red-disk-parameterized stimuli are shown for (b) the Scintillating Grid illusion experiment and (c) the No Bars experiment.

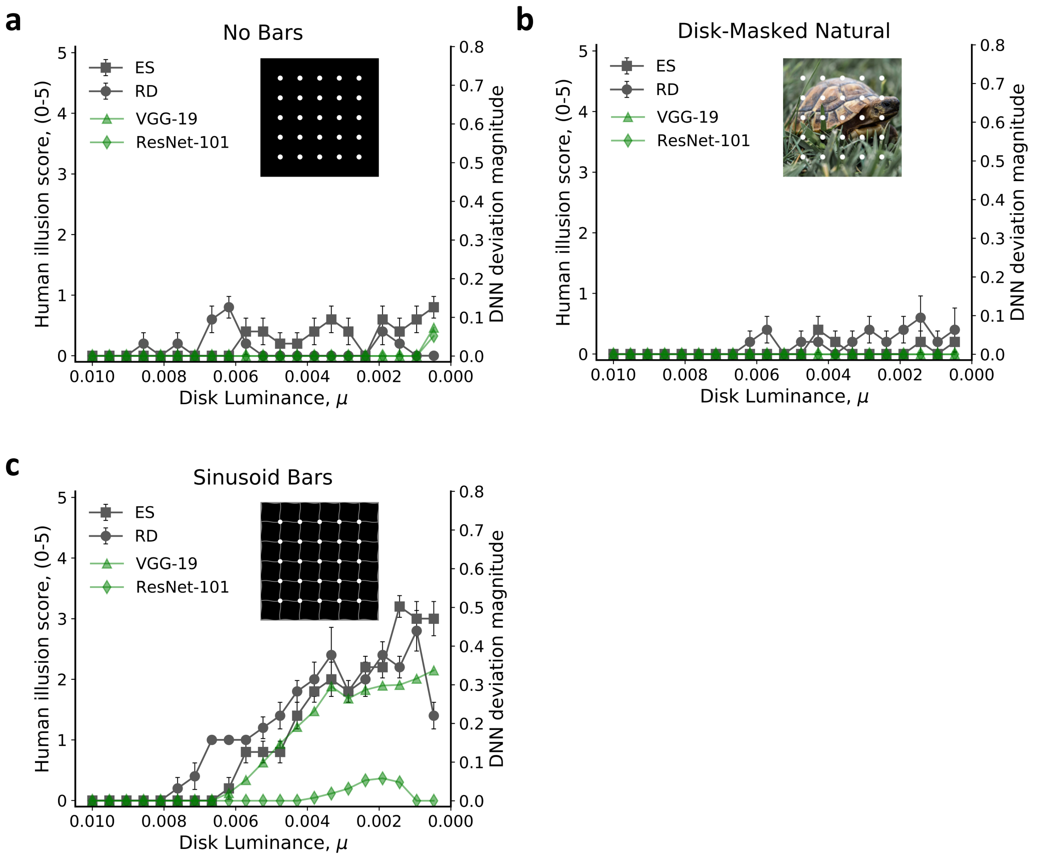

Figure S7: Comparison to human perception for non-illusion control images. Same analysis as in Fig. 4b for (a) one No Bars control image, (b) one natural image with disk masking (here, the example natural image is represented by a public-domain image instead of ImageNet images), and (c) one Sinusoid Bars reduced illusion control image. Results for the No Bars and Disk-masked natural image controls show close to zero reported illusion scores for both human observers and close to zero deviation magnitudes for both DNNs. The Sinusoid Bars image had positive illusion scores for humans and positive deviation magnitudes for DNNs but not the same level as the original Scintillating Grid (see Fig .4b).

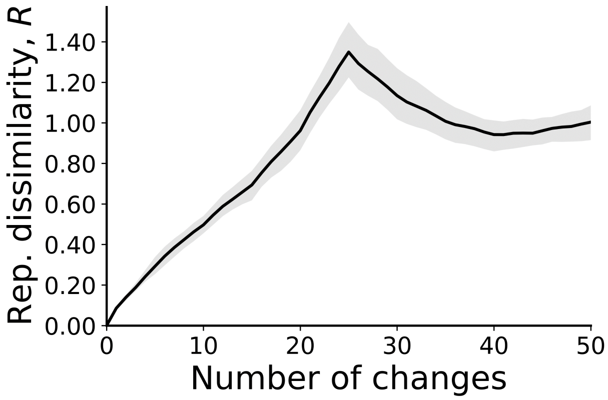

Figure S8: Representational dissimilarity with disks changed from white to middle gray to black. The changes progress with each of the white disks being changed to middle gray ($\mu = 0.5$) and then each of the middle gray disks being changed to black.

Appendix B: Controlling for a non-monotonicity due to contrast or shape loss

As seen in the main Results section, the relation between representational dissimilarity and disk luminance in the Scintillating Grid shows a peak in deviation magnitudes around $\mu = 0.5$, which is the cause of the observed non-monotonicity (Fig. 2a). A plausible explanation of this peak is that around $\mu = 0.5$, the disk luminance is most similar to the bar luminance, leading to loss of contrast and/or disk contours and results in maximal dissimilarity with the reference image, which has intact disk contrast and contour. To dissociate this hypothesis from the predictions based on illusion perception, we considered manipulations of disk luminance in images with a gray (luminance 0.5) background and no bars (Fig. S10a). In these Gray Background images, there was no illusion effect when $\mu = 1$, and also complete loss of shape/contrast when $\mu = 0.5$ with the absence of the disk border. Therefore, if the non-monotonicity is mediated by shape/contrast loss, we expect stronger non-monotonicity for the Gray Background as compared to the Scintillating Grid. If, however, the non-monotonicity indicates illusion-like response, we expect the opposite. In this manipulation we also considered the width of the border around the disks to be 0, 1, 2, or 3 pixels (in the main text, all experiments used a one-pixel disk border). For increased border width, there should be a decreased loss of shape around $\mu = 0.5$, consistent with the results showing a sharp peak in the representational dissimilarity at $\mu = 0.5$ for Gray Background images with no disk border, and a less pronounced peak for larger border widths (see Fig. SS9). Consequently, the manipulation of border width provides an additional tool to dissociate between the illusion perception and the shape loss hypotheses.

Gray Background images with no disk border produced significantly larger deviation magnitudes than the corresponding images with the standard one-pixel disk border in both VGG-19 and ResNet-101 (see gray line in Fig. S10bc). This suggests that the presence of a disk border greatly reduces the effect of contrast or shape loss. In comparison, the Scintillating Grid images produced slightly higher deviation magnitudes in the presence of the one-pixel disk border (see green line in Fig. S10bc). This difference in the dependence of deviation magnitude on disk border indicates that the non-monotonic deviation from the Scintillating Grid cannot be fully explained by loss of contrast or shape contours (as in the Gray Background control). Furthermore, these results suggest that a one-

pixel disk border is able to preserve the illusion-like representation in DNNs while greatly reducing the non-monotonic effect of contrast or shape loss.

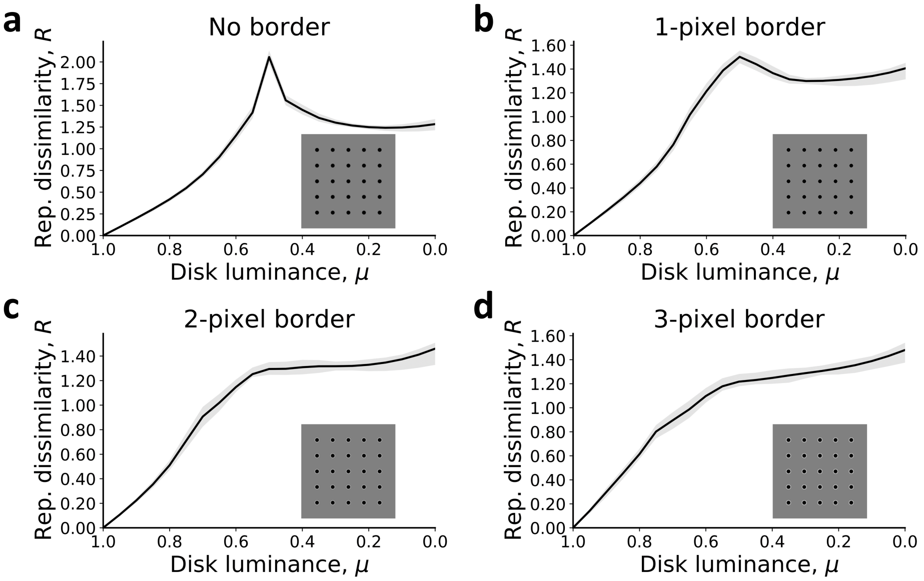

Figure S9: Mean representational dissimilarity for the Gray Background control image set when disk border width is (a) 0 pixels, (b) 1 pixel, (c) 2 pixels, (d) 3 pixels. Insets show example Gray Background images with $\mu = 0$. A sharply non-monotonic peak is observed near $\mu = 0.5$ for the unbordered gray background image in panel a, and the peak depresses with increasing disk border width. Shaded region corresponds to the interquartile range. Note that the y-axis scaling is different in the different panels.

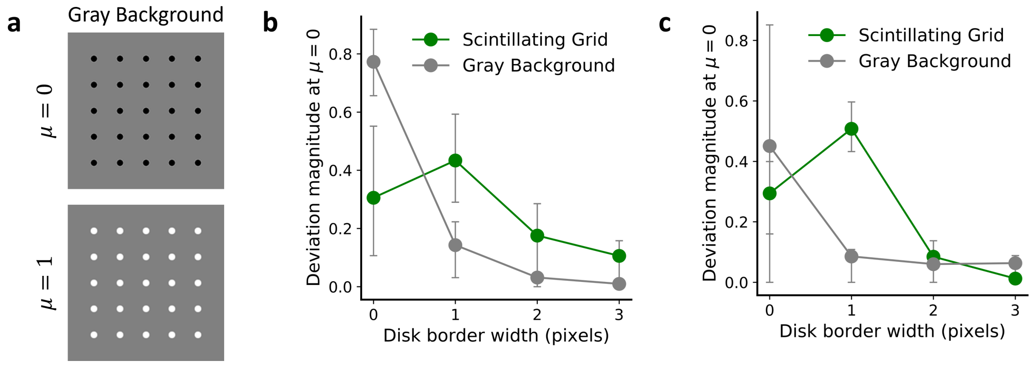

Figure S10: Deviation magnitudes for Gray Background and Scintillating Grid images across different border widths. (a) Example Gray Background images with $\mu = 0$ (top) and $\mu = 1$ (bottom). In the experimental manipulation, the disk luminance is increased from white to black, as in the experiments reported in Figs. 2-4. (b-c) Mean deviation magnitudes at $\mu = 0$ for the Gray Background images (in gray) and the Scintillating Grid images (in green) for different border widths (see Methods); (b) for VGG-19; the data point of the Scintillating Grid for border width 1 is reproduced from Fig. 3c; (c) for ResNet-101; the data point of the Scintillating Grid for border width 1 is reproduced from Fig. S5c. Error bars correspond to the interquartile range.

A dissociation between the illusion perception and shape loss hypotheses was also evident when considering the different computational stages (Fig. S11). Overall, comparing Gray Background to Scintillating Grid, deviation magnitudes at $\mu = 0$ were usually highest in mid-early layers for Gray Background, but highest in the late layers for Scintillating Grid.

When there was no disk border (Fig. S11ac), deviation magnitudes at $\mu = 0$ were typically higher in the Gray Background than in Scintillating Grid. For VGG-19 (Fig. S11a), the Gray Background showed deviation magnitudes that were first positive at layer conv3_1 and soon reached a maximum at conv3_3, while the Scintillating Grid was positive first at conv3_2 with a gradual increase thereafter (we refer to the layer outputs after ReLU). Importantly, the conv3_1, conv3_2, and conv3_3 convolutions have receptive field sizes of 24X24, 32X32, and 40X40 pixels respectively. Therefore, considering that the used disks were of size 9, 11, or 13 pixels, the initial increase for both Gray Background and Scintillating Grid corresponds to double of the disk size. For ResNet-101, measured deviation magnitudes roughly resembled those of VGG-19 (compare Fig. S11a to Fig. S11c).

For the one-pixel disk border images, the relationship is reversed: the Scintillating Grid images have higher deviation magnitudes than the Gray Background images (Fig. S11bd). In both DNNs, the deviation magnitude of one-pixel border images gradually increases across deeper computational stages for Scintillating Grid images and is constantly low for Gray Background images. For VGG-19 (Fig. S11b), the first layer to have positive deviation magnitude was conv3_2 for Scintillating Grid and conv3_1 for Gray Background (for the border-absent case). The computational stage with maximum deviation magnitude was conv5_2 for Scintillating Grid and conv3_3 for Gray Background, similar to the border-absent case. It can be observed that high deviation magnitudes for the Gray Background were almost only found at the convolutions with receptive field sizes two to four times the disk size, arguably in line with contrast detectors. For ResNet-101, results were roughly similar to VGG-19 (compare Fig. S11b to Fig. S11d).

These differences in the deviation magnitudes across the DNN computational stage hierarchy suggest that the one-pixel disk border significantly reduces the contribution of contrast and shape loss to deviation magnitude and either preserves or augments illusion-specific deviation magnitude. Altogether, these findings support the conclusion that the observed non-monotonicity in DNN representations of the Scintillating Grid cannot be fully explained by contrast/shape loss.

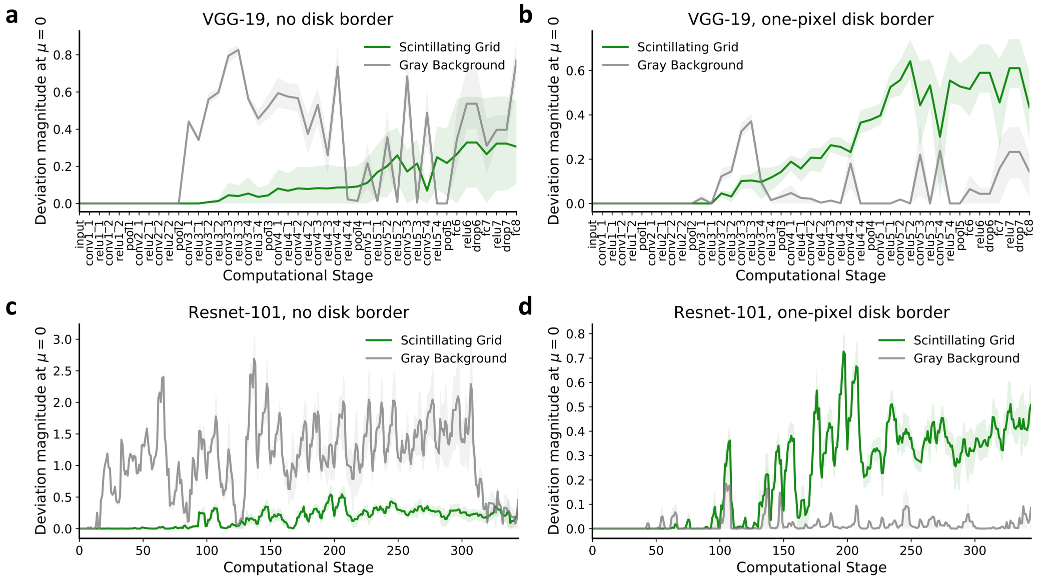

Figure S11: Comparing the Gray Background and the Scintillating Grid across the DNN computational hierarchy. Shown are the mean deviation magnitudes at $\mu = 0$ for Scintillating Grid (green) and Gray Background (gray) images; (a) for VGG-19 and images with no disk border; (b) for VGG-19 and images with one-pixel disk border; (c) for ResNet-101 and images with no disk border; (d) for ResNet-101 and images with one-pixel disk border. The Scintillating Grid data is reproduced from Fig. 4. Shaded regions represent the interquartile range. Note that the y-axis scaling is different in the different panels.

Appendix C: Non-monotonicity in human response to Scintillating Grid stimuli

Although the experiments in Fig. 5c and Fig. SS7 provide an intuitive setup to compare human and DNN responses across different stimuli sets, the design of these experiments has two important weaknesses: (1) the measure "human illusion score" is subjective in nature, and (2) the measure does not directly test for non-monotonicity of representational dissimilarity with disk luminance in humans. To address these concerns, we designed a second experiment in which the same subjects are briefly shown an image for 0.5 seconds that is either an unaltered Scintillating Grid image or an altered image with seven of eight peripheral disks uniformly changed to a non-white luminance ($\mu < 1.0$) (see Fig. SS12a for examples). After the stimuli disappears, a masking stimulus consisting of random grayscale noise with the same dimensions is shown for 1.0 second. The subject then discriminates whether the image is an unaltered image or an altered image using the arrow keys on a keyboard. The correctness of the decision and the response time is recorded for each trial. In total, each subject was presented with 336 image stimuli (168 unaltered, 168 distinct altered stimuli) in random order. All other environmental and experimental parameters were identical to the earlier human experiments (see Methods section) with the exception of the monitor illuminance when the stimuli was absent, which was 0-20 lux in this experiment.

In this experiment, the accuracy saturated for most tasks for $\mu < 0.4$ (see Fig. S12bc). As a result, we focused on the median response times for correct discrimination trials as a proxy for representational distance (i.e. longer response times correspond to greater discrimination difficulty

and thus smaller representational distance from the unaltered Scintillating Grid). For both subjects, the response times typically decreased monotonically with decreased disk luminance in the No Bars control experiment (Fig. S12e). In the Scintillating Grid experiment, there was clear non-monotonic response times with disk luminance for one subject and no significant non-monotonic response times for the other subject. In the latter case, the lack of non-monotonicity is likely due to low signal-to-noise ratio from the low difficulty in the discrimination task (as evidenced by the saturating response time). To resolve these effects better, we computed the normalized response time difference $= \mathrm{RT}_{\mathrm{Illusion}} - \mathrm{RT}_{\mathrm{No\ Bars}}$, where RT is the median response times normalized by the median response time at $\mu = 1.0$. The normalized response time difference typically increased as the disk luminance decreased for $\mu < 0.55$, which suggests that there is increasing difficulty observed for the Scintillating Grid (presumably due to illusion perception) than for the No Bars control. Altogether, these results support our earlier findings of similar responses to the Scintillating Grid between human and DNNs.

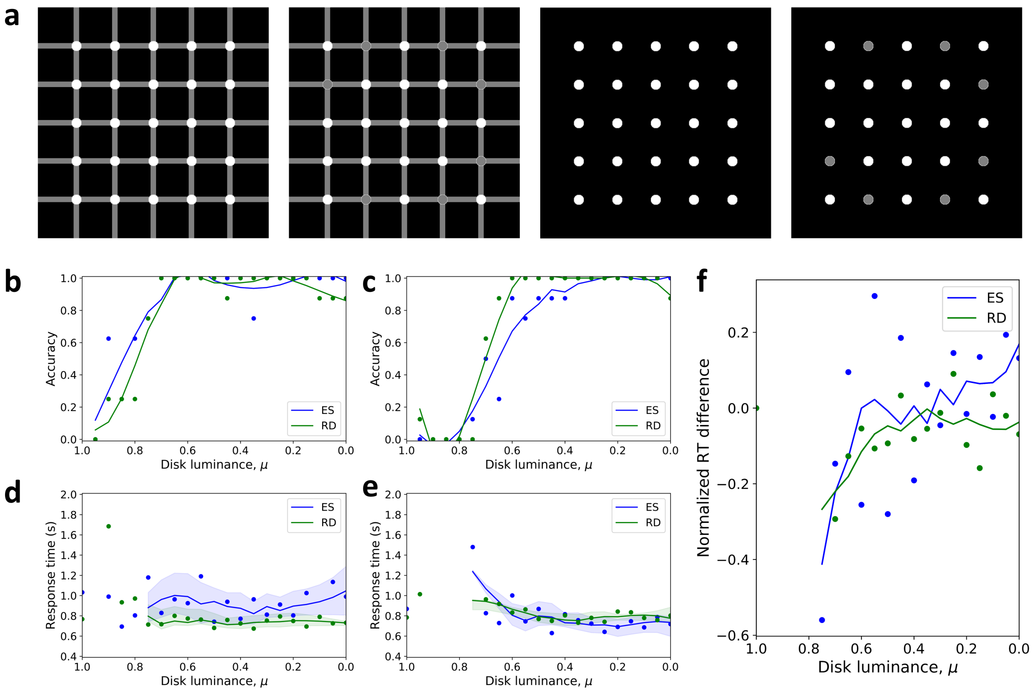

Figure S12: Human discrimination of altered Scintillating Grid stimuli. (a) Shown are examples of altered and unaltered images; from left to right: unaltered Scintillating Grid, altered Scintillating Grid, unaltered No Bars, altered No Bars. In plots (b-c), the points correspond to accuracy at each luminance level and in plots (d-e), the median response times for correct answers at each luminance level. (f) Normalized response time difference between Scintillating Grid and No Bars tasks across different luminance levels. In all plots, the lines correspond to smoothed trends and the shaded regions correspond to the smoothed interquartile range.
